# MDMA-induced changes in within-network connectivity contradict the specificity of these alterations for the effects of serotonergic hallucinogens

**DOI:** 10.1038/s41386-020-00906-2

**Published:** 2020-11-20

**Authors:** Felix Müller, Friederike Holze, Patrick Dolder, Laura Ley, Patrick Vizeli, Alain Soltermann, Matthias E. Liechti, Stefan Borgwardt

**Affiliations:** 1 Department of Psychiatry (UPK), University of Basel, Basel, 4002 Switzerland; 2 Division of Clinical Pharmacology and Toxicology, Department of Biomedicine and Department of Clinical Research, University Hospital Basel, University of Basel, Basel, 4031 Switzerland; 3 Department of Psychiatry and Psychotherapy, University of Lübeck, Lübeck, 23538 Germany

**Keywords:** Translational research, Neuroscience

## Abstract

It has been reported that serotonergic hallucinogens like lysergic acid diethylamide (LSD) induce decreases in functional connectivity within various resting-state networks. These alterations were seen as reflecting specific neuronal effects of hallucinogens and it was speculated that these shifts in connectivity underlie the characteristic subjective drug effects. In this study, we test the hypothesis that these alterations are not specific for hallucinogens but that they can be induced by monoaminergic stimulation using the non-hallucinogenic serotonin–norepinephrine–dopamine releasing agent 3,4-methylenedioxymethamphetamine (MDMA). In a randomized, placebo-controlled, double-blind, crossover design, 45 healthy participants underwent functional magnetic resonance imaging (fMRI) following oral administration of 125 mg MDMA. The networks under question were identified using independent component analysis (ICA) and were tested with regard to within-network connectivity. Results revealed decreased connectivity within two visual networks, the default mode network (DMN), and the sensorimotor network. These findings were almost identical to the results previously reported for hallucinogenic drugs. Therefore, our results suggest that monoaminergic substances can induce widespread changes in within-network connectivity in the absence of marked subjective drug effects. This contradicts the notion that these alterations can be regarded as specific for serotonergic hallucinogens. However, changes within the DMN might explain antidepressants effects of some of these substances.

## Introduction

Several recent studies have assessed how serotonergic hallucinogens exert their typical effects on the brain [1]. This also comprised studies which applied fMRI [2]. One focus of these investigations was the impact of hallucinogenic drugs like LSD and ayahuasca on functional connectivity (FC) of resting-state networks (RSN) [3–5]. Overall, it was found that these substances induce widespread decreases in FC within several networks [2]. Based on these observations, the hypothesis was formulated that hallucinogens act by compromising integrity within RSNs and that these alterations explain some of the profound effects of these substances on the psyche [4].

Notably, it has been reported that the selective serotonin reuptake inhibitor (SSRI) sertraline induces changes in within-network FC which closely resemble those seen after LSD [4–6]. While hallucinogens induce profound mental changes like positive mood, visual alterations, and loss boundaries of the self [7, 8], SSRIs only cause very minor alterations such as mild impairment of learning and cognition [9] or affective flattening [10]. Although these substances are therefore clearly distinct on the level of subjective effects it is obvious that they share monoaminergic stimulation as a common factor. Prototypical hallucinogens like LSD and psilocybin mainly act through agonism at the serotonin_2A_-receptor [11], a mechanism which mediates all typical subjective drug effects [12, 13]. To some extent, hallucinogens also interact with the dopamine system [11]. The SSRI sertraline inhibits reuptake of serotonin, but also interacts with the dopamine transporter [14].

We have already hypothesized elsewhere that the observed alterations in within-network FC after administration of hallucinogens might be an epiphenomenon, most likely induced by unspecific serotonergic stimulation and therefore possibly not specific or explanatory for the effects of these drugs [2, 5]. In accordance with this hypothesis, it has not yet been possible to reliably link alterations in within-network FC to subjective hallucinogenic drug effects. Solely, a significant association between decreases in FC within the DMN  and the subjective experience of “ego dissolution” has been described [4], but replication has not been possible so far [5].

Besides changes in within-network FC, it has also been reported that the SSRI escitalopram induces increases in degree centrality in thalamic and cerebellar regions [15]. Degree centrality is a measure for the number of “connections” of a given node within a network [16]. It has been described that the hallucinogens psilocybin and LSD acutely increase degree centrality in thalamic and other regions and these alterations have also been linked to the mechanism of action of hallucinogens [17–19]. Relating these findings to the results described above, it was speculated that hallucinogens generally act by decreasing FC within several networks but increase FC between regions of distinct networks [4, 17]. These opposing effects might be induced by recruitment of heterogeneous populations of inhibitory and excitatory cells [20]. However, given the observation after administration of an SSRI [15] there is the possibility that these findings might also represent unspecific serotonergic effects.

Taken together, there is some preliminary evidence that fMRI findings that were interpreted as reflecting typical hallucinogenic drug effects [3–5, 17, 18] might actually be induced by unspecific monoaminergic stimulation. So far, however, this hypothesis is solely based on two individual studies investigating the effects of SSRIs [6, 15]. The aim of the present study was to further investigate this possibility by using MDMA, a potent serotonin–norepinephrine-dopamine releasing agent with a diverging mechanism of action [21] and a clearly distinct profile of subjective drug effects [22].

## Methods

This analysis is based on pooled data sets of two clinical trials (ClinicalTrials.gov identifier: NCT01951508 and NCT03019822). Both studies were conducted in Basel (Switzerland) and approved by the Ethics Committee for Northwest/Central Switzerland (EKNZ) and by the Federal Office of Public Health. All subjects gave written consent prior to participating and received monetary compensation.

### Study design

Both pooled studies tested the effects of different psychoactive substances in healthy participants using a randomized, placebo-controlled, double-blind, crossover design. Study 1 tested the effects of MDMA, methylphenidate, modafinil, and placebo and study 2 tested the effects of MDMA, LSD, d-amphetamine, and placebo. These trials were almost identical. Both studies included a prescreening telephone interview, a screening visit, four experimental sessions, and an end-of-study visit. Washout periods between sessions were at least 7 (study 1) and 10 days (study 2), respectively. MDMA hydrochloride (Lipomed AG, Arlesheim, Switzerland) was prepared as gelatin capsules with mannitol as filler. Identical capsules were prepared as placebo. MDMA was administered as a single oral dose of 125 mg. Administration took place at 9:45 a.m. (study 1) or 9:30 a.m. (study 2). The MRI scan was performed between 11:15 and 12:15 a.m. (study 1) or between 11:00 and 12:00 a.m. (study), taking into account the expected drug peak effects [23, 24]. Both studies included identical assessments of subjective drug effects, physiological parameters, and plasma drug concentrations. More details are reported elsewhere [22, 25].

### Participants

Participants were recruited by advertisement on the University of Basel website and by word of mouth. Inclusion criteria were age between 18 and 45 years, sufficient understanding of the German language, and a body mass index between 18 and 27 kg/m^2^ (study 1) or 18 and 29 kg/m^2^ (study 2), respectively. Subjects were excluded if they met at least one of the following criteria: acute or chronic medical condition, hypertension or hypotension, current or previous major psychiatric diseases, psychotic diseases in first-degree relatives, prior illicit drug use of >5 lifetime episodes (study 1) or >10 lifetime episodes (study 2) or any time within the last 2 months (except tetrahydrocannabinol), contraindications for MRI, participation in another clinical trial (currently or within the last month), use of drugs that are contraindicated or interfere with study drugs, tobacco smoking (>10 cigarettes/day), and pregnancy or nursing. Prior to each study day, participants had to provide urine samples to rule out illicit drug use. In addition, women underwent a pregnancy test.

Overall, 52 participants received 125 mg MDMA or placebo (study 1: 24 participants; study 2: 28 participants). After exclusion of seven subjects (please see below) the final sample consisted of 45 participants (22 women, 23 men; mean age: 26.2 years ±SD 4.4). Eleven of these participants had used MDMA before (1–5 times), 12 had used stimulants (1–4 times), five had used hallucinogens (1–2 times), seven had used sedatives (1–5 times), and one subject had used an opioid (one time). Cannabis had been used by 38 participants (19: 1–9 times, 11: 10–19 times, 8: ≥20 times).

### Image acquisition

The imaging data were collected using a 3 Tesla MRI system (Magnetom Prisma, Siemens Healthcare, Erlangen, Germany) with a 20-channel phased-array radio frequency head coil. Functional MRI acquisition was based on an interleaved T2*-weighted echo-planar imaging sequence. The following parameters were used: 35 axial slices with a slice thickness of 3.5 mm, a 0.5 mm inter-slice gap, a field-of-view of 224 × 224 cm^2^, and an in-plane image matrix size of 64 × 64—resulting in 3.5 × 3.5 × 3.5 mm^3^ resolution. The repetition time was 1.8 s, echo time 28 ms and bandwidth = 2442 Hz/pixel. During the scan, participants were briefed to close their eyes and remain awake. Three hundred volumes were acquired for each condition.

### Preprocessing

Data were processed and analyzed using the CONN toolbox 19c (http://www.nitrc.org/projects/conn) [26] based on SPM12 (http://www.fil.ion.ucl.ac.uk/spm/) running in MATLAB 2019a. Before quality assessment, fMRI data were available for 51 participants (one participant was excluded a priori because this person completed the resting-state sequences with eyes open).

The first five volumes of the functional time series were not considered in order to ensure magnetization equilibrium. Preprocessing of the functional images included realignment (registration to the first image of the time series; 2nd-degree b-spline interpolation), unwarping (4th-degree b-spline interpolation), slice-time correction, direct segmentation into gray and white matter and cerebrospinal fluid (using the default SPM tissue probability maps), normalization into a standard stereotactic space (Montreal Neurological Institute; MNI), and smoothing (5 mm full width at half maximum Gaussian kernel). Noise correction of the functional images included scrubbing with a global signal threshold of *z* > 3 and a composite subject motion threshold of >0.5 mm using ART as implemented in CONN, linear detrending, and linear regression of the six motion parameters.

Pharmacological fMRI studies are prone to biases induced by the respective pharmacological agent, like alterations in physiological parameters or in neurovascular coupling [27]. CompCor is an approach to remove physiological fluctuations by extracting principal components from regions unlikely to be modulated by neural activity and then including these components as nuisance parameters [28]. Following this approach, five principal components were extracted from white matter and cerebrospinal fluid signals (using individual tissue masks obtained from the T_1_-weighted structural images) and removed using CompCor as implemented in CONN [28].

The resulting functional images were band-pass filtered (0.008 < *f* < 0.09 Hz) as previous evidence suggests that this procedure improves independent component results in addition to high-pass filtering [29].

Quality assessment comprised three stages: First, all scans were assessed considering the percentage of scrubbed volumes. Subjects were excluded if <5 min of the scan remained after scrubbing (corresponding to <55% of the initial volumes). This was due to evidence indicating that resting-state scans <5 min are not reliable [30]. One subject was excluded based on this criterium. The mean percentage of scrubbed volumes for the drug condition was 6.3% (±SD 3.2) and 4.8% (±SD 2.0) for the placebo condition. Second, mean and maximal head motion after the scrubbing procedure were assessed using the measure framewise displacement (FD) which was calculated according to Power et al. [31]. A sphere radius of 50 mm was chosen for the calculation. Subjects were excluded if maximum FD was >0.75 mm (half-voxel size). Six subjects were excluded based on this criterium, resulting in a final sample of 45 participants.

Last, mean and maximum FD after scrubbing were tested for significant differences between conditions. All data were plotted, visually inspected, and tested (Shapiro–Wilk test) to assess normality distribution and this prerequisite was met for all data. Values between drug and placebo conditions were compared using paired *t* tests and significance was assumed at *p* < 0.05, uncorrected. Mean FD after scrubbing was 0.14 mm (±SD 0.05) for MDMA and 0.15 mm (±SD 0.04) after placebo. Average maximum FD after scrubbing was 0.62 mm (±SD 0.28) and 0.50 (±SD 0.21), respectively. There was no significant difference for mean FD (*p* = 0.33) but average maximum FD was significantly higher under the drug condition (*p* = 0.02).

Therefore, potential influences of this measure on FC [32] were further explored (please see below).

### Independent component analysis

ICA was performed using group-ICA procedures implemented in the CONN toolbox which follow methods described by Calhoun et al. [33].

ICA results are determined by the chosen number of dimensions, i.e., a higher number of dimensions might result in a higher number of distinct resting-state networks compared with a lower number of dimensions. ICA was restricted to 20 factors in order to allow comparisons with 10 established resting-state network described by Smith et al. [34]. The same restriction was used in previous studies on hallucinogens [4, 5]. Furthermore, ICA analysis comprised dimensionality reduction on the subject-level (64 dimensions), temporal concatenation across all subjects and both conditions, group-level dimensionality reduction (20 components), and G1 FastICA for the estimation of independent components. Subject-level maps were estimated using GICA3 back-projection.

Estimation of subject-specific components in group-ICA is limited by a trade-off between a group model and subject-specific representations of these components. Regarding this estimation, GICA3 back-projection was found to be superior compared with other GICA back-projections and compared with dual regression [35].

Decisions regarding the labeling of the networks identified in this data set were based on visual inspection [36] and cross-correlation of the unthresholded ICA components with the unthresholded resting-state networks described by Smith et al. (https://www.fmrib.ox.ac.uk/datasets/brainmap+rsns/).

### Within-network functional connectivity

Analysis of within-network FC followed the same steps as previously described [5]: Ten unthresholded ICA components identified as RSNs in the ICA analysis were compared between the drug and the placebo condition (paired *t* tests). A voxel threshold of *p* < 0.001 (uncorrected) and a cluster-size threshold of *p* < 0.005 (Bonferroni corrected; FWE), which also accounted for testing 10 different components (*p* < 0.05/10 = *p* < 0.005), was used. Thresholded maps (*z* = 2) of these networks were used to assess whether significant clusters fall within the respective network.

### Degree centrality

Degree centrality is a measure for FC between a given node and the rest of the brain. There are different ways to calculate degree centrality, e.g., by averaging all correlation coefficients between a voxel and all other voxels or by thresholding or squaring these values prior to calculation. For calculation of degree centrality in this study, the preprocessed images were imported in DPABI 4.3 [37]. Degree centrality was then calculated in the same way as described by Schaefer et al. in order to allow comparisons with their analysis on the effects of an SSRI [15]. This procedure comprised the following steps: The ICBM152 gray matter mask [38] was thresholded at >25% and further analysis was restricted to this mask. For each voxel, FC between this voxel and all other voxels across the brain was calculated. The derived correlation coefficients were thresholded at *r* > 0.15 and summed up for each voxel. The resulting images were *z*-transformed (the mean was subtracted and images were divided by standard deviations). Conditions were compared by a two-tailed paired *t* test (SPM12 running in MATLAB 2019a). A voxel threshold of *p* < 0.001 (uncorrected) and a cluster-size threshold of *p* < 0.05 (Bonferroni corrected; FWE) was applied.

### Influence of changes in physiological parameters and head motion on functional connectivity

It has already been shown, that MDMA increases heart rate, systolic and diastolic blood pressure, and body temperature [22, 25]. It is also known that all these measures might bias fMRI results [27, 39]. Therefore, potential influences of these measures on FC results were assessed.

All physiological parameters just described were taken right before (1.5 h after administration) and after the MRI (2.5 h after administration) [22, 25]. Both time points were averaged for all parameters because the resting-state sequence took place in the middle of the scan. In the first step, it was estimated whether there were significant differences between conditions for these parameters. Values were missing for two subjects at one time point (2.5 h after administration) in the placebo condition and for one subject at both time points (1.5 and 2.5 h after administration in the MDMA condition). These subjects were excluded. All data were plotted, visually inspected, and tested (Shapiro–Wilk test) in order to assess normality distribution. This prerequisite was not met for all parameters. Therefore, Wilcoxon tests were used. Significance was assumed at *p* < 0.05 (two-tailed). All physiological parameters were elevated under the drug condition and differed significantly compared with placebo (all *p* < 0.001; mean difference systolic blood pressure: 21.6 mmHg ± SD 11.9; mean difference diastolic blood pressure: 11.5 mmHg ± SD 9.2; mean difference heart rate: 16.5/min ± SD 10.2; mean difference body temperature: 0.3 degree ± SD 0.4). Therefore, all parameters were tested for potential confounding effects on FC.

Confounding was assessed using two different approaches. First, possible associations were tested by correlation analyses. FC values of all significant clusters (∆FC = FC_MDMA_ − FC_Placebo_) derived in our analyses were extracted and correlated with changes in maximum FD (∆FD = FD_MDMA_ − FD_Placebo_) and changes in physiological parameters (∆PP = PP_MDMA_ − PP_Placebo_). Spearman’s rho was used because not all data were normally distributed. Cases of missing values were treated as described above. Results were reported both uncorrected (*p* < 0.10) and corrected for multiple testing (*p* < 0.10, FWE; Bonferroni). The same analysis was applied to test the influence of average maximum FD (see above) on FC result.

Second, confounding was assessed by entering the demeaned values of all parameters (∆FD = FD_MDMA_ − FD_Placebo_ and ∆PP = PP_MDMA _− PP_Placebo_) as covariates of no interest in the second-level analyses. The same statistical thresholds as in the original analyses were used (ICA analysis: voxel threshold of *p* < 0.001 (uncorrected), cluster-size threshold *p* < 0.005, FWE; degree centrality analysis: voxel threshold *p* < 0.001 (uncorrected), cluster-size threshold of *p* < 0.05, FWE).

### Qualitative comparison with other monoaminergic drugs

MDMA, LSD, and sertraline primarily work by stimulation of the serotonin system [11, 14, 21]. However, all three substances also stimulate the dopamine system to some extent [11, 14, 40, 41] and MDMA also interacts with the noradrenaline system [11]. We performed a systematic literature search to investigate the possibility that results obtained in this and other fMRI studies might be attributable to other neurotransmitter systems than serotonin. The PubMed database was searched for fMRI studies investigating the effects of serotonergic, dopaminergic, and noradrenergic drugs on resting-state networks in healthy subjects (date of search: 17 September 2020). Results were screened for studies investigating relevant networks [4–6] derived with ICA. We used the following search term: fMRI AND (serotonin OR dopamine OR noradrenaline OR norepinephrine) AND (ICA OR “independent component analysis” OR networks OR network). Studies were then qualitatively compared with regard to the neurotransmitter profiles of the respective substance.

## Results

### Independent component analysis

There was a good agreement between most of the components identified in our analysis and the templates provided by Smith et al. [34]. We were able to identify the visual networks 1–3 (*r* = 0.76, *r* = 0.72, *r* = 0.66, respectively), the cerebellar network (*r* = 0.24), the auditory network (*r* = 0.41), the frontoparietal network 1 and 2 (*r* = 0.25 and *r* = 0.43), and the executive control network (*r* = 0.40). The DMN only consisted of the posterior part of the network (*r* = 0.56) and we were not able to reliably identify the anterior part in our data set. Similar representations of the DMN and the sensorimotor network in ICA analyses have already been described in several studies (e.g., [42, 43]) and were also used in the studies under question [4, 5]. All ten networks identified in our data set are shown in Supplementary Figure 1.

### Within-network connectivity

**Fig. 1:**
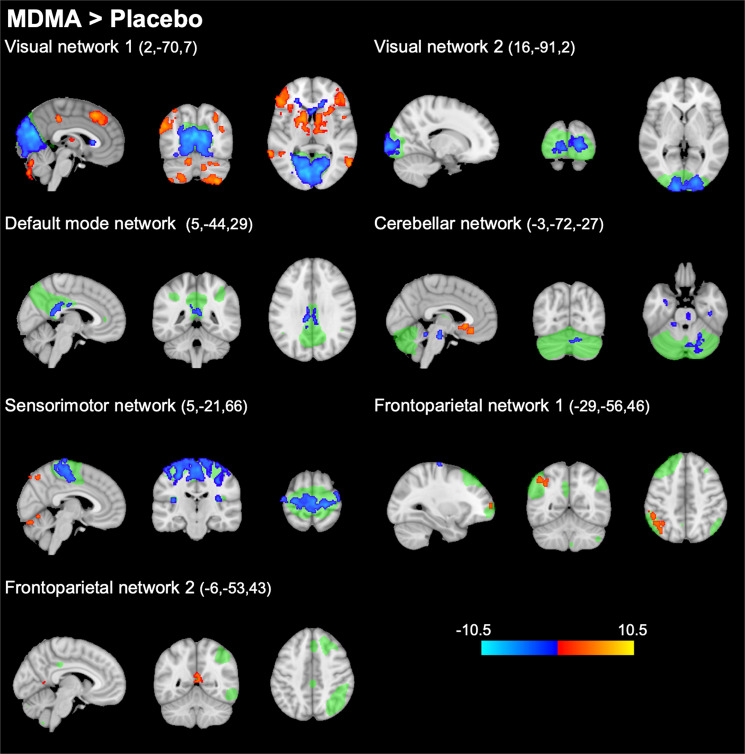
Alterations in within-network functional connectivity (FC) after administration of MDMA compared with placebo (thresholded at *p* < 0.005, FWE, on the basis of a cluster-forming threshold of *p* < 0.001). Resting-state networks identified in our data set are shown in green. MDMA significantly decreased FC within several networks (shown in blue). MDMA increased FC within parts of the frontoparietal networks (shown in red). After adjustment for potential confounds, alterations within the cerebellar network were no longer significant. These findings are nearly identical to alterations described after the administration of the hallucinogen LSD [4, 5]. The colorbar indicates the *t* values. *X*, *Y*, and *Z* values indicate MNI coordinates. Right is the right side of the brain.

Compared with placebo, MDMA decreased FC within the visual networks 1 and 2, the DMN, the cerebellar network, and the sensorimotor network (*p* < 0.005, FWE, on the basis of a cluster-forming threshold of *p* < 0.001). Decreases in FC were most pronounced for the visual networks 1 and 2 and for the sensorimotor network where large portions of the respective network showed altered FC. Alterations in the DMN were more discrete, where FC was decreased within a smaller cluster located in the medial-posterior part of the DMN (posterior cingulate gyrus). Increases in FC were found in the frontoparietal networks 1 and 2. There were no significant alterations in within-network FC for the visual network 3, the auditory network, and the executive control network. Please see Fig. 1 and Supplementary Table 1 for more details. Supplementary Figure 2 shows a qualitative comparison of findings in this study and studies on LSD [4, 5].

### Degree centrality

**Fig. 2:**
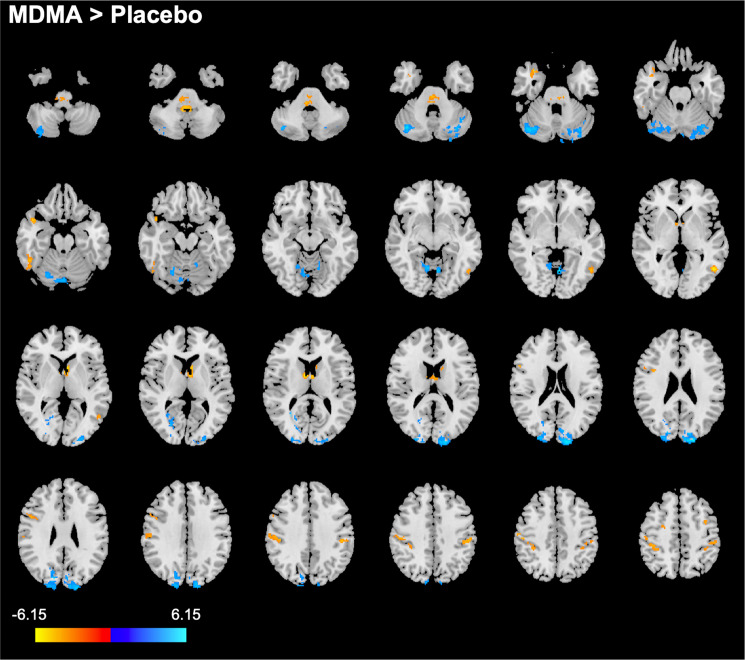
Alterations in degree centrality after administration of MDMA compared with placebo (*p* < 0.05, FWE, on the basis of a cluster-forming threshold of *p* < 0.001). Increases in degree centrality are shown in yellow-red, decreases are shown in blue. After adjustment for potential confounds, none of these results remained significant. The colorbar indicates the *t* values. Right is right side of the brain.

Compared with placebo, MDMA decreased degree centrality in the cerebellum and in occipital regions (mostly occipital pole, cuneal, intracalcarine, and lingual cortex). Degree centrality was increased in several cortical regions (mostly post- and precentral gyrus, supramarginal gyrus, superior parietal lobule, middle frontal gyrus, superior frontal gyrus, temporal gyrus, temporal pole, lateral occipital cortex), and the brain-stem. Increases were also observed in a cluster located in the most anterior part of the thalamus and parts of the left caudate. Results of this analysis are shown in Fig. 2. More details are reported in Supplementary Table 2.

### Influence of head motion and changes in physiological parameters on functional connectivity

Using an uncorrected threshold of *p* < 0.10, there was no evidence that maximal FD or any of the physiological parameters (heart rate, blood pressure, body temperature) were systemically associated with FC measures in our ICA analyses or in our degree centrality analysis (please see Supplementary Figure 3). None of these correlations reached significance after correction for multiple comparisons (*p* < 0.10, FWE).

In line with this, entering maximal FD and physiological parameters as covariates of no interest did not significantly alter the results of the ICA analysis (please see Supplementary Figure 4). However, this was not true for the cerebellar network where within-network FC was no longer significantly decreased after correction.

In contrast, inclusion of covariates had a great impact on the degree centrality analysis, where no significant differences between MDMA and placebo were observed after adjustment.

### Qualitative comparison with other monoaminergic drugs

Our literature search yielded nine fMRI studies which investigated effects of levodopa [44, 45], haloperidol [45], escitalopram [46], methylphenidate [47], d-amphetamine [48], bupropion [49], tryptophan depletion/loading [50], and citalopram [51]. One study reported decreased FC within ten of ten investigated networks after citalopram, but it was not reported whether these findings were significant [52]. Results of all selected studies and studies on the effects of sertraline [6], LSD [4, 5], and MDMA (this study) are shown in Table 1. Most of the networks affected by sertraline, LSD, and MDMA were not investigated by other studies. However, all studies investigated the (posterior) DMN. The table also depicts interactions with the neurotransmitter systems under question [11, 14, 40, 41, 53–57].

**Table 1:** Alterations in within-network functional connectivity (FC) in this work qualitatively compared with studies on other serotonergic, dopaminergic, and norepinephrinergic drugs.

Blue cells indicate decreases and red cells indicate increases in within-network FC. Gray cells indicate no alterations, black cells indicate that the network was not investigated. Impacts on neurotransmitter systems are shown using the same color scheme. This table refers to results in [4–6, 44–52] and findings in this study.

^a^Result was no longer significant after adjustment for potential confounds. 5-HT: serotonin, dopa: dopamine, NA: noradrenaline, L-dopa: levodopa, TRP: tryptophan.

^b^Increased FC was reported in a small cluster at the borderline of the network.

## Discussion

We tested the acute effects of the potent serotonin releaser MDMA on within-network FC and degree centrality. If our hypothesis were true, then the non-hallucinogenic drug MDMA should induce similar alterations as already described for SSRIs [6, 15] and hallucinogenic drugs [3–5]. Overall, this hypothesis was confirmed with regard to within-network FC, but not with regard to degree centrality. Therefore, our data do not support the notion that alterations in within-network FC can be regarded as explanatory for the typical acute effects of hallucinogenic drugs, as previously proposed [4, 5].

More specifically, MDMA acutely decreased FC within two visual networks, the default mode network (DMN), the sensorimotor network, and the cerebellar network. These alterations were not systemically associated with several potential confounders (head motion, changes in heart rate, blood pressure, and body temperature). However, this was not true for the cerebellar network where no significant alterations were observed after correction. Actually, this finding is in line with previous studies on sertraline and LSD, where this network was not affected [4–6]. Overall, our results are in good agreement with these studies: Across all substances (SSRI, LSD, MDMA), decreases in FC were observed for the visual network 1, the medial-posterior part of the DMN, and the sensorimotor network [4–6]. Our results are also in line with a study on the hallucinogen ayahuasca which exclusively investigated the DMN [3]. Another consistent finding was that FC was not altered within the auditory network and the executive network. However, results were not entirely consistent across studies for other visual networks and for the frontoparietal networks.

Although changes in within-network FC do not seem to be specific for acute hallucinogenic effects, this does not exclude the possibility that these alterations might be important for other domains, e.g., therapeutic long-term effects of these substances [58–60]. Probably the most obvious finding in this regard refers to decreased FC in the posterior DMN which was observed in this study and after administration of LSD [4, 5]. Depression has been repeatedly linked to increased FC within the DMN, including the posterior part of this network (for an overview please see Sundermann et al. [61]). It has also been demonstrated that different antidepressants such as escitalopram [62], duloxetine [63], sertraline [6], and ketamine [64, 65] decrease FC within the DMN. Among others, all these studies reported decreased FC within the posterior cingulate cortex, a finding which was also seen after ayahuasca [3], LSD [4, 5], and after MDMA in this study. According to a recent model, hallucinogens might induce lasting positive effects by acute destabilization of pathological connectivity patterns and consecutive establishment of new connectivity patterns [66]. Acutely decreased FC within the posterior DMN might represent such an effect and might thus explain some long-lasting effects of psilocybin [67], LSD [58], and possibly also MDMA [68].

In contrast to within-network FC, we did not find evidence that MDMA induced similar changes in degree centrality as reported for the SSRI escitalopram, where widespread decreases but local increases in cerebellum and thalamus were reported [15]. In our study, degree centrality was decreased in cerebellar regions and minor changes were observed in a cluster covering small portions of the thalamus and parts of the left caudate. However, none of these results remained significant after inclusion of covariates. This might indicate that these findings were induced by changes in physiological parameters or head motion after MDMA.

Overall, our study underlines known obstacles in pharmacological fMRI, namely the presence of findings induced by unspecific drug effects or various sources of confounders [27, 39]. Such factors might also bias other findings than within-network FC. Several studies have indicated that administrations of hallucinogens were also associated with distinct increases in FC between various networks [2]. Based on these observations, the hypothesis was formulated that hallucinogens act by compromising integrity within RSNs while blurring demarcations between them and that these alterations explain the profound effects of these substances on the psyche [4]. However, very little agreement was found in more detail, i.e., regarding FC between individual RSNs [2]. Given these difficulties in identifying clear neuronal correlates of hallucinogenic drug effects, it might be advantageous to directly relate subjective drug effects to fMRI data, e.g., using regression analyses. Moreover, known confounders should be addressed more consistently. The present analysis demonstrates that comparison with related psychoactive substances might also be worthwhile.

This study has several limitations: First, we do not provide dose-response data. Typically, one would expect that alterations in brain activity induced by psychoactive substances are dose-dependent. However, if the findings observed in this and other studies were induced by monoaminergic stimulation, it would be unclear how equivalent doses across different substances could be defined (e.g., it is unclear how an equivalent dose of the serotonin agonist LSD compared with the serotonin releaser MDMA could be defined in terms of serotonergic stimulation). Furthermore, although MDMA does not induce typical hallucinogenic drug effects [22] there are some overlaps as “empathogenic” effects associated with MDMA are also induced by LSD to some extent [8]. However, the networks under question are typically not associated with these effects. Moreover, sample sizes and statistical thresholds varied across studies and there are additional differences. These factors limit comparison between studies. Global pharmacological effects like alterations in physiological parameters or in neurovascular coupling might have biased results. Our analysis comprised post hoc procedures to exclude such global effects. However, regression based on continuous measures of physiological parameters or direct tests of alterations in neurovascular coupling might have been favorable.

It should also be noted that low-dimensional ICA as applied in this study provides relatively large networks. It is possible that hallucinogenic specific effects within subnetworks might be detectable using high-dimensional approaches. Moreover, different measures of degree centrality were used across studies [15, 17, 18]. In this study, the measure used by Schaefer et al. [15] was chosen because of the greatest explanatory power for our analysis.

Although stimulation of the serotonin system is the main mechanism of action of MDMA, LSD, and the SSRI sertraline, it should be noted that all of these substances have some affinity for other neurotransmitter receptors or transporters. Namely, all three substances stimulate the dopamine system to some extent [11, 40, 41] and MDMA additionally simulates the noradrenaline system [11]. Therefore, the observed effects on within-network FC might be mediated through other systems than the serotonin system, particularly the dopamine system. In order to investigate this possibility, comparison with studies on other monoaminergic drugs might be helpful. However, studies identified in our literature search did not investigate most of the networks under question, except for DMN. Regarding the DMN, findings were inconclusive. In addition, neurotransmitter profiles of the investigated substances were mostly ambiguous. Therefore, the question whether alterations in within-network FC might be induced by stimulation of the dopamine system remains open.

In summary, this study demonstrated that it is possible to induce very similar alterations in within-network FC seen after administration of LSD by using the non-hallucinogenic drug MDMA. The alterations observed in this study were not associated with potential sources of bias. These results are generally in line with findings described after intake of an SSRI and query the specificity and explanatory value of these alterations for the effects of hallucinogenic drugs. It remains to be resolved whether these alterations are induced by serotonergic or dopaminergic stimulation.

## Funding and disclosure

This work was supported by the Swiss National Science Foundation (grant no. 32003B_185111 to MEL and grant no. 320030_170249 to MEL and SB). MEL acts as a consultant to Mind Medicine Inc. The other authors declare no conflicts of interests. Know-how and data associated with this work and owned by the University Hospital Basel has been licensed by Mind Medicine Inc. after study completion. Mind Medicine had no role in the financing, planning, and conduct of the present study or the present publication. Open Access funding provided by University of Basel.

## Supplementary information

Related Manuscript File

Supplemental Material

CONSORT flowchart
