# Supplementary material for: MDMA-induced changes in within-network connectivity contradict the specificity of these alterations for the effects of serotonergic hallucinogens
Source: Neuropsychopharmacology. 2020 Nov 20;46(3):545–53. doi: 10.1038/s41386-020-00906-2 (PMC8027447; doi:10.1038/s41386-020-00906-2)
Supplement: Supplementary file 1 — Related Manuscript File [file 41386_2020_906_MOESM1_ESM.ppt]

## Slide 1
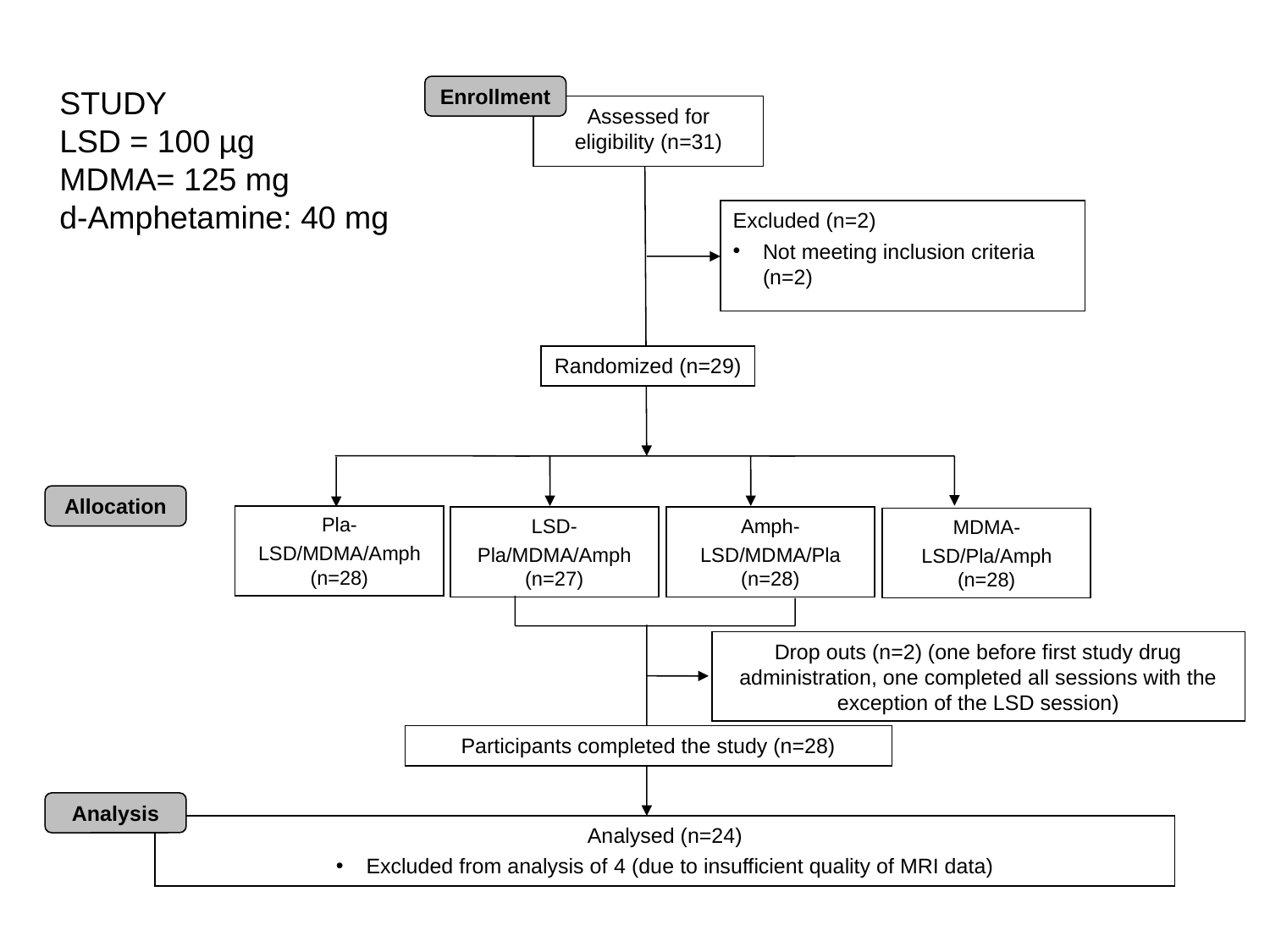

STUDY
LSD = 100 µg
MDMA= 125 mg
d-Amphetamine: 40 mg
Enrollment
# Assessed for eligibility (n=31)
Excluded (n=2)
Not meeting inclusion criteria (n=2)
Randomized (n=29)
Allocation
Pla-
LSD/MDMA/Amph (n=28)
LSD-
Pla/MDMA/Amph (n=27)
Amph-
LSD/MDMA/Pla (n=28)
MDMA-
LSD/Pla/Amph (n=28)
Drop outs (n=2) (one before first study drug administration, one completed all sessions with the exception of the LSD session)
Participants completed the study (n=28)
Analysis
Analysed (n=24)
Excluded from analysis of 4 (due to insufficient quality of MRI data)
