# Supplementary material for: MDMA-induced changes in within-network connectivity contradict the specificity of these alterations for the effects of serotonergic hallucinogens
Source: Neuropsychopharmacology. 2020 Nov 20;46(3):545–53. doi: 10.1038/s41386-020-00906-2 (PMC8027447; doi:10.1038/s41386-020-00906-2)
Supplement: Supplementary file 2 — Supplemental Material [file 41386_2020_906_MOESM2_ESM.pdf]

Supplemental information

MDMA-induced changes in within-network connectivity contradict the specificity of these alterations for the effects of serotonergic hallucinogenic drugs

Felix Müller MD[1*], Friederike Holze MSc[2], Patrick Dolder PhD[2], Laura Ley MSc[2], Patrick Vizeli PhD[2], Alain Soltermann BSc[1], Matthias E. Liechti MD[2], Stefan Borgwardt MD[1]

[1]University of Basel, Department of Psychiatry (UPK), Basel 4012, Switzerland, [2]University of Basel, Division of Clinical Pharmacology and Toxicology, Department of Biomedicine and Department of Clinical Research, University Hospital Basel, Basel 4031, Switzerland

Supplementary Figures

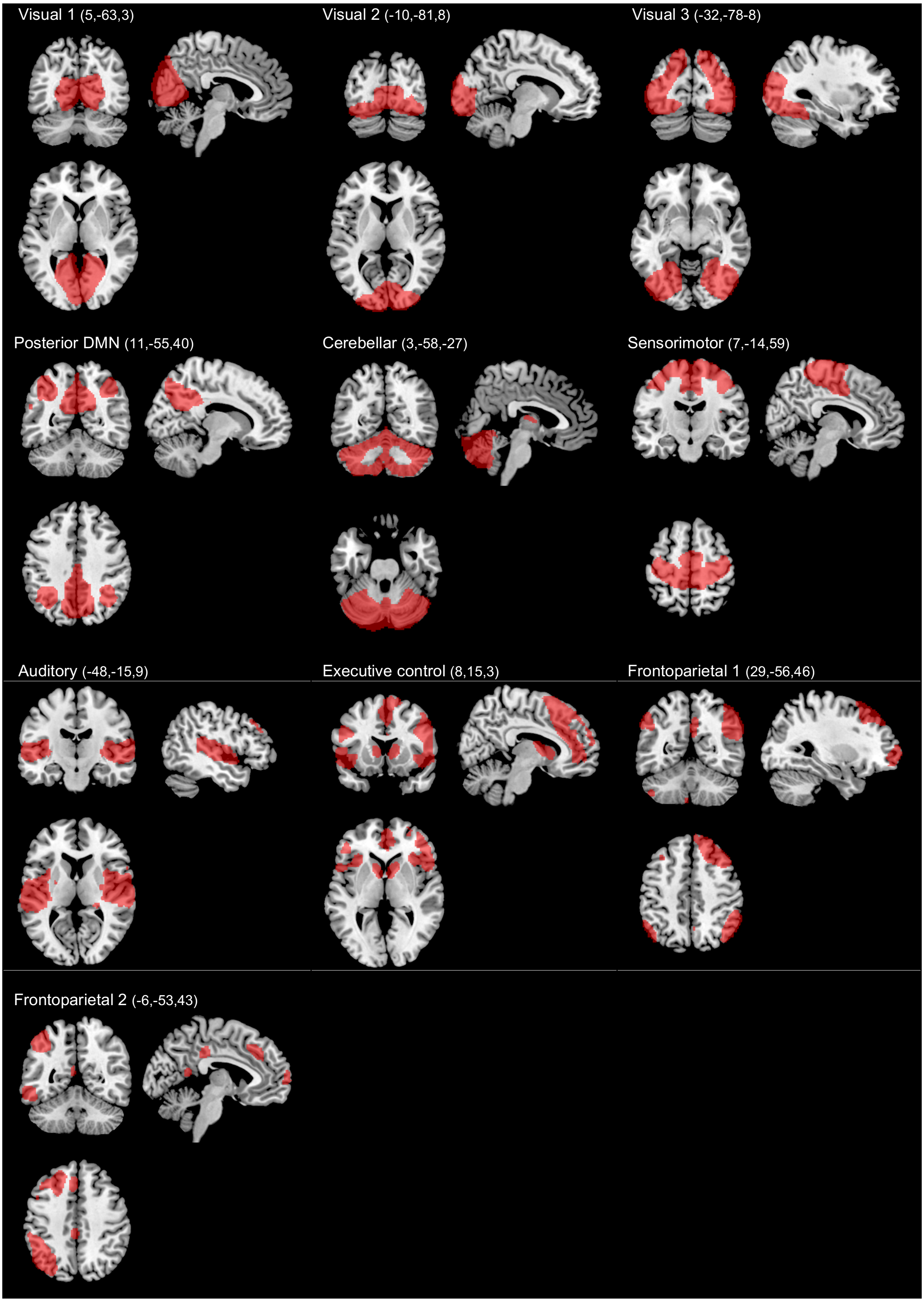

Supplementary figure 1: Resting state networks identified in this study (shown in red). Networks were labeld according to Smith et al. [1]. Images are thresholded at z≥2. Values indicate MNI coordinates (X,Y,Z). Images are shown in radiological convention (right is left).

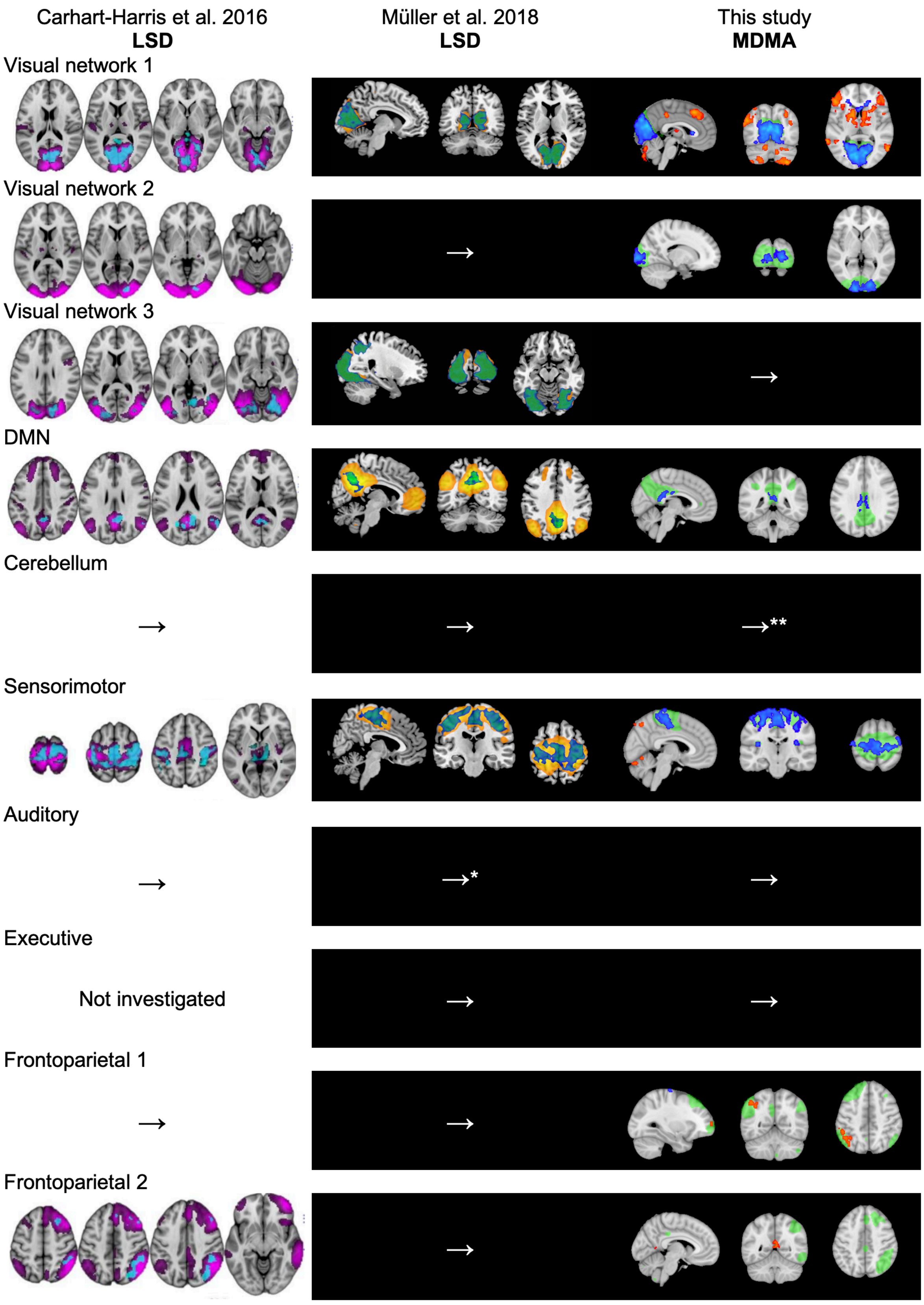

Supplementary figure 2: Qualitative Comparison of within-network functional connectivity (FC) in studies discussed in this publication. Horizontal arrows indicate no alterations compared with placebo. Resting state networks are shown in purple (Carhart-Harris et al.), yellow (Müller et al. 2018), and green (this study), respectively. Decreases in within-network FC are shown in blue (Carhart-Harris et al., this study) and green (Müller et al. 2018), respectively. Increases are shown in red. *Increased FC was reported in a small cluster at the borderline of the network.**Result was no longer significant after adjustment for potential confounds Figures reprinted from: [2] and [3]. Right is the right side of the brain.

A: ICA-analyses

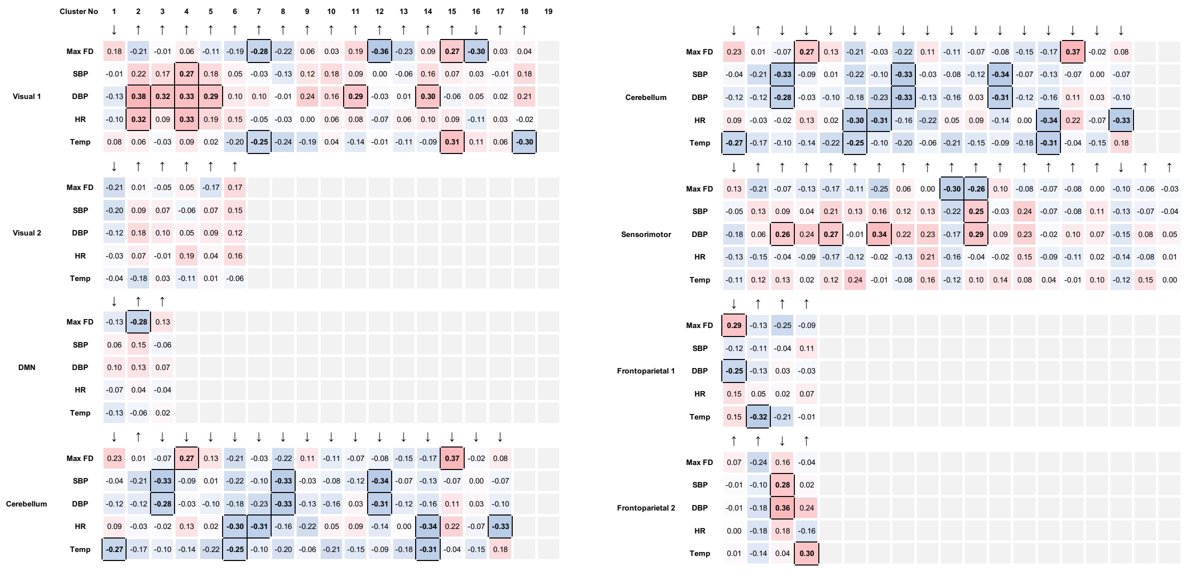

B: Degree centrality analysis

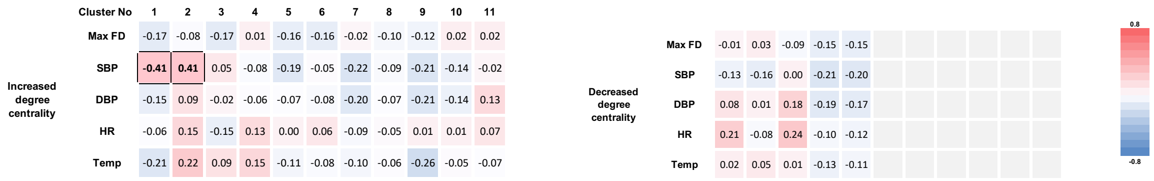

Supplementary figure 3: Correlations between changes in (**A**) network connectivity / (**B**) degree centrality and potential confounds (max FD: maximum framewise displacement, SBP: systolic blood pressure, DBP: diastolic blood pressure, HR: heart rate, Temp: body temperature). The colorbar indicates the correlations coefficient (Spearman’s rho). Positive correlations are shown in red, negative correlations are shown in blue. Arrows indicate increases and decreases in FC in the respective cluster. Significant correlations at a threshold of p<0.10 uncorrected for multiple comparisons are highlighted. None of these correlations remained significant after correction for multiple comparisons (p<0.10, FWE).

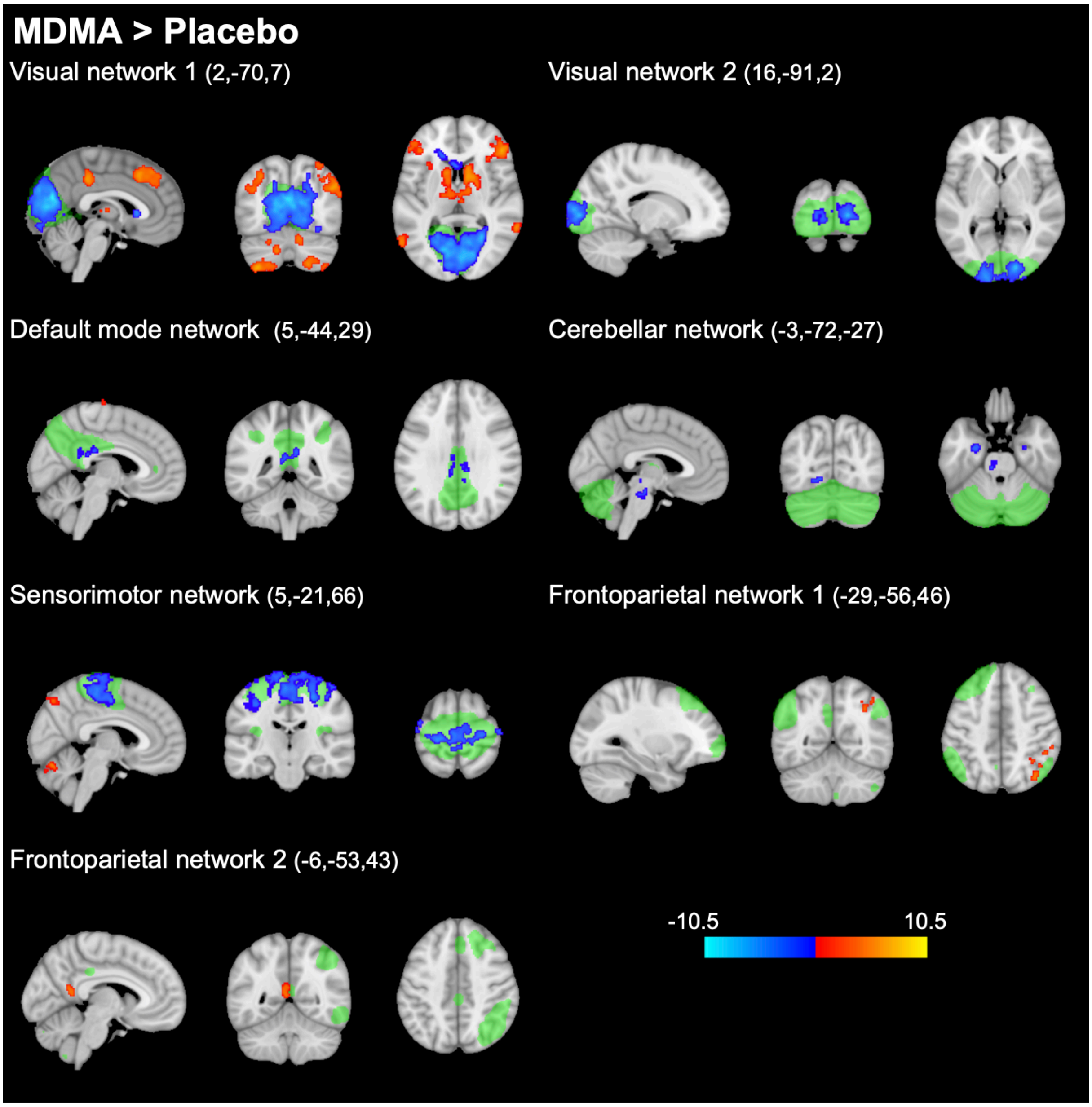

Supplementary figure 4: Alterations in within-network functional connectivity (FC) after administration of MDMA compared with placebo after inclusion of several covariates of no interest (covariates: changes in systolic and diastolic blood pressure, heart rate, body temperature, and maximal framewise displacement). Resting state networks identified in our data set are shown in green, decreases in FC are shown in blue, increases in FC are shown in blue. Inclusion of covariates did not significantly alter the results of the main ICA analysis. However, this was not true for the cerebellar network where within-network FC was no longer significantly decreased after correction.Images are thresholded using the same threshold as in our main analysis ($p<0.005$, FWE, on the basis of a cluster-forming threshold of $p<0.001$) and the same coordinates are shown.

Supplementary Tables

Anatomical regions are reported according to the Harvard-Oxford cortical and subcortical structural atlas [4] and the Automated anatomical labeling atlas for the cerebellum [5].

Table 1

Cluster no.	MNI			Size (voxels)	p value FWE (cluster size)	p value uncorrected (cluster size)	Brain regions (percentage covered by cluster)
	x	y	z				
Within-network functional connectivity							
**Visual 1**							
**1**	6	-80	8	10717	<0.000001	<0.000001	1488 voxels covering 86% of Lingual Gyrus R<br>1323 voxels covering 87% of Lingual Gyrus L<br>856 voxels covering 15% of Precuneous Cortex<br>750 voxels covering 100% of Intracalcarine Cortex R<br>641 voxels covering 100% of Cuneal Cortex R<br>640 voxels covering 100% of Intracalcarine Cortex L<br>548 voxels covering 21% of Occipital Pole L<br>515 voxels covering 99% of Cuneal Cortex L<br>455 voxels covering 18% of Occipital Pole R<br>223 voxels covering 5% of Lateral Occipital Cortex, superior division R<br>180 voxels covering 4% of Lateral Occipital Cortex, superior division L<br>143 voxels covering 100% of Supracalcarine Cortex R<br>120 voxels covering 13% of Cerebellum 4 5 L<br>98 voxels covering 4% of Cingulate Gyrus, posterior division<br>97 voxels covering 8% of Cerebellum 6 L<br>86 voxels covering 13% of Temporal Occipital Fusiform Cortex L<br>78 voxels covering 10% of Temporal Occipital Fusiform Cortex R<br>66 voxels covering 90% of Supracalcarine Cortex L<br>63 voxels covering 7% of Occipital Fusiform Gyrus R<br>59 voxels covering 10% of Cerebellum 4 5 R<br>36 voxels covering 4% of Occipital Fusiform Gyrus L<br>33 voxels covering 5% of Vermis 4 5<br>27 voxels covering 2% of Cerebellum 6 R<br>10 voxels covering 3% of Vermis 6<br>4 voxels covering 1% of Parahippocampal Gyrus, posterior division L<br>2178 voxels covering 1% of not-labeled
**2**	58	-64	24	2624	<0.000001	<0.000001	1503 voxels covering 31% of Lateral Occipital Cortex, superior division R<br>450 voxels covering 31% of Angular Gyrus R<br>253 voxels covering 20% of Supramarginal Gyrus, posterior division R<br>125 voxels covering 8% of Superior Parietal Lobule R<br>7 voxels covering 0% of Lateral Occipital Cortex, inferior division R<br>2 voxels covering 0% of Supramarginal Gyrus, anterior division R<br>284 voxels covering 0% of not-labeled
**3**	50	22	20	2458	<0.000001	<0.000001	1058 voxels covering 39% of Middle Frontal Gyrus R<br>289 voxels covering 11% of Superior Frontal Gyrus R<br>266 voxels covering 3% of Frontal Pole R<br>218 voxels covering 39% of Inferior Frontal Gyrus, pars triangularis R<br>198 voxels covering 29% of Inferior Frontal Gyrus, pars opercularis R<br>83 voxels covering 2% of Precentral Gyrus R<br>346 voxels covering 0% of not-labeled
**4**	-32	-76	40	2196	<0.000001	<0.000001	1328 voxels covering 27% of Lateral Occipital Cortex, superior division L<br>248 voxels covering 23% of Supramarginal Gyrus, posterior division L<br>221 voxels covering 15% of Superior Parietal Lobule L<br>88 voxels covering 9% of Supramarginal Gyrus, anterior division L<br>57 voxels covering 6% of Angular Gyrus L<br>9 voxels covering 0% of Postcentral Gyrus L<br>245 voxels covering 0% of not-labeled

**5**	-56	-48	-10	1020	<0.000001	<0.000001	423 voxels covering 49% of Middle Temporal Gyrus, temporooccipital part L<br>168 voxels covering 24% of Inferior Temporal Gyrus, temporooccipital part L<br>127 voxels covering 9% of Middle Temporal Gyrus, posterior division L<br>126 voxels covering 6% of Lateral Occipital Cortex, inferior division L<br>10 voxels covering 1% of Inferior Temporal Gyrus, posterior division L<br>4 voxels covering 0% of Angular Gyrus L<br>162 voxels covering 0% of not-labeled
**6**	66	-46	4	1019	<0.000001	<0.000001	580 voxels covering 50% of Middle Temporal Gyrus, temporooccipital part R<br>180 voxels covering 23% of Inferior Temporal Gyrus, temporooccipital part R<br>59 voxels covering 4% of Middle Temporal Gyrus, posterior division R<br>19 voxels covering 1% of Lateral Occipital Cortex, inferior division R<br>13 voxels covering 1% of Angular Gyrus R<br>3 voxels covering 0% of Inferior Temporal Gyrus, posterior division R<br>1 voxels covering 0% of Supramarginal Gyrus, posterior division R<br>164 voxels covering 0% of not-labeled
**7**	12	16	2	818	<0.000001	<0.000001	213 voxels covering 41% of Caudate r<br>179 voxels covering 22% of Putamen r<br>59 voxels covering 22% of Pallidum r<br>41 voxels covering 3% of Thalamus r<br>18 voxels covering 21% of Accumbens r<br>17 voxels covering 5% of Amygdala r<br>1 voxels covering 0% of Frontal Orbital Cortex R<br>290 voxels covering 0% of not-labeled
**8**	-10	4	2	623	<0.000001	<0.000001	217 voxels covering 25% of Putamen l<br>131 voxels covering 24% of Caudate l<br>30 voxels covering 2% of Thalamus l<br>21 voxels covering 7% of Pallidum l<br>21 voxels covering 6% of Amygdala l<br>14 voxels covering 13% of Accumbens l<br>4 voxels covering 0% of Insular Cortex L<br>3 voxels covering 1% of Planum Polare L<br>182 voxels covering 0% of not-labeled
**9**	4	22	42	567	<0.000001	<0.000001	178 voxels covering 13% of Paracingulate Gyrus R<br>127 voxels covering 5% of Superior Frontal Gyrus R<br>79 voxels covering 3% of Superior Frontal Gyrus L<br>66 voxels covering 5% of Paracingulate Gyrus L<br>5 voxels covering 0% of Cingulate Gyrus, anterior division<br>112 voxels covering 0% of not-labeled
**10**	-24	12	54	530	<0.000001	<0.000001	295 voxels covering 10% of Superior Frontal Gyrus L<br>193 voxels covering 7% of Middle Frontal Gyrus L<br>42 voxels covering 0% of not-labeled
**11**	-54	10	36	482	<0.000001	<0.000001	170 voxels covering 22% of IFG Inferior Frontal Gyrus, pars opercularis L<br>157 voxels covering 5% of Middle Frontal Gyrus L<br>86 voxels covering 2% of Precentral Gyrus L<br>69 voxels covering 0% of not-labeled
**12**	-14	-76	-28	334	<0.000001	<0.000001	173 voxels covering 9% of Cerebellum Crus2 L<br>92 voxels covering 4% of Cerebellum Crus1 L<br>20 voxels covering 4% of Cerebellum 7b L<br>3 voxels covering 2% of Vermis 7<br>1 voxels covering 0% of Cerebellum 8 L<br>45 voxels covering 0% of not-labeled
**13**	-34	-72	-56	321	<0.000001	<0.000001	78 voxels covering 4% of Cerebellum 8 L<br>72 voxels covering 13% of Cerebellum 7b L<br>8 voxels covering 0% of Cerebellum Crus2 L<br>163 voxels covering 0% of not-labeled
**14**	-46	30	18	317	<0.000001	<0.000001	189 voxels covering 29% of Inferior Frontal Gyrus, pars triangularis L<br>59 voxels covering 2% of Middle Frontal Gyrus L<br>35 voxels covering 1% of Frontal Pole L<br>6 voxels covering 0% of Frontal Orbital Cortex L<br>28 voxels covering 0% of not-labeled

**15**	-16	30	6	233	0.000009	<0.000001	233 voxels covering 0% of not-labeled
**16**	6	-34	40	221	0.000015	<0.000001	207 voxels covering 9% of Cingulate Gyrus, posterior division<br>14 voxels covering 0% of not-labeled
**17**	12	-72	-24	205	0.000031	0.000001	99 voxels covering 5% of Cerebellum Crus2 R<br>42 voxels covering 3% of Cerebellum 6 R<br>33 voxels covering 1% of Cerebellum Crus1 R<br>28 voxels covering 5% of Cerebellum 7b R<br>2 voxels covering 0% of Cerebellum 8 R<br>1 voxels covering 0% of not-labeled
**18**	34	26	4	119	0.002413	0.000047	43 voxels covering 3% of Insular Cortex R<br>41 voxels covering 3% of Frontal Orbital Cortex R<br>22 voxels covering 7% of Frontal Operculum Cortex R<br>13 voxels covering 0% of not-labeled
**Visual 2**							
**1**	18	-90	2	4868	<0.000001	<0.000001	1559 voxels covering 62% of Occipital Pole R<br>1538 voxels covering 58% of Occipital Pole L<br>231 voxels covering 25% of Occipital Fusiform Gyrus L<br>159 voxels covering 18% of Occipital Fusiform Gyrus R<br>143 voxels covering 9% of Lingual Gyrus L<br>131 voxels covering 8% of Lingual Gyrus R<br>110 voxels covering 15% of Intracalcarine Cortex R<br>68 voxels covering 11% of Intracalcarine Cortex L<br>43 voxels covering 2% of Lateral Occipital Cortex, inferior division L<br>30 voxels covering 1% of Lateral Occipital Cortex, inferior division R<br>5 voxels covering 0% of Cerebellum Crus1 L<br>4 voxels covering 3% of Supracalcarine Cortex R<br>3 voxels covering 0% of Lateral Occipital Cortex, superior division R<br>2 voxels covering 0% of Cerebellum 6 L<br>842 voxels covering 0% of not-labeled
**2**	-4	16	-6	347	<0.000001	<0.000001	135 voxels covering 25% of Caudate l<br>63 voxels covering 6% of Subcallosal Cortex<br>16 voxels covering 19% of Accumbens r<br>15 voxels covering 14% of Accumbens l<br>11 voxels covering 1% of Thalamus l<br>1 voxels covering 0% of Caudate r<br>106 voxels covering 0% of not-labeled
**3**	18	-42	-14	172	0.000081	0.000001	108 voxels covering 6% of Lingual Gyrus R<br>55 voxels covering 9% of Cerebellum 4 5 R<br>7 voxels covering 0% of Precuneous Cortex<br>2 voxels covering 0% of Temporal Occipital Fusiform Cortex R
**4**	50	-12	-40	163	0.00013	0.000002	80 voxels covering 25% of Inferior Temporal Gyrus, anterior division R<br>58 voxels covering 6% of Inferior Temporal Gyrus, posterior division R<br>2 voxels covering 0% of Temporal Fusiform Cortex, posterior division R<br>23 voxels covering 0% of not-labeled
**5**	0	-20	44	115	0.001909	0.000035	80 voxels covering 3% Cingulate Gyrus, posterior division<br>6 voxels covering 0% of Precentral Gyrus R<br>3 voxels covering 0% of Cingulate Gyrus, anterior division<br>26 voxels covering 0% of not-labeled
**6**	-42	26	42	108	0.002907	0.000053	108 voxels covering 4% of Middle Frontal Gyrus L
**DMN**							

**1**	8	-28	28	516	<0.000001	<0.000001	273 voxels covering 11% of Cingulate Gyrus, posterior division<br>1 voxels covering 0% of AC Cingulate Gyrus, anterior division<br>242 voxels covering 0% of not-labeled
**2**	-24	-2	-14	242	0.000002	<0.000001	103 voxels covering 14% of Hippocampus l<br>78 voxels covering 24% of Amygdala l<br>32 voxels covering 4% of Putamen l<br>5 voxels covering 2% of Pallidum l<br>24 voxels covering 0% of not-labeled
**3**	-48	22	38	102	0.003347	0.000059	102 voxels covering 3% of Middle Frontal Gyrus L
**Cerebellum**							
**1**	-26	0	-4	474	<0.000001	<0.000001	249 voxels covering 29% of Putamen l<br>91 voxels covering 17% of Caudate l<br>30 voxels covering 10% of Pallidum l<br>104 voxels covering 0% of not-labeled
**2**	4	20	-4	403	<0.000001	<0.000001	176 voxels covering 16% of Subcallosal Cortex<br>2 voxels covering 0% of Frontal Medial Cortex<br>225 voxels covering 0% of not-labeled
**3**	-24	-68	-26	400	<0.000001	<0.000001	212 voxels covering 9% of Cerebellum Crus1 L<br>131 voxels covering 10% of Cerebellum 6 L<br>29 voxels covering 2% of Cerebellum Crus2 L<br>14 voxels covering 7% of Vermis 7<br>7 voxels covering 0% of Cerebellum 8 L<br>2 voxels covering 1% of Vermis 8<br>5 voxels covering 0% of not-labeled
**4**	28	2	-24	345	<0.000001	<0.000001	195 voxels covering 24% of Putamen r<br>56 voxels covering 16% of Amygdala r<br>9 voxels covering 3% of Pallidum r<br>4 voxels covering 1% of Hippocampus r<br>81 voxels covering 0% of not-labeled
**5**	-18	-40	-24	323	<0.000001	<0.000001	69 voxels covering 8% of Cerebellum 4 5 L<br>68 voxels covering 11% of Cerebellum 4 5 R<br>43 voxels covering 19% of Vermis 3<br>24 voxels covering 1% of Brain-Stem<br>24 voxels covering 4% of Vermis 4 5<br>19 voxels covering 1% of Cerebellum 6 L<br>18 voxels covering 10% of Cerebellum 3 R<br>10 voxels covering 8% of Cerebellum 3 L<br>4 voxels covering 1% of Vermis 6<br>1 voxels covering 0% of Parahippocampal Gyrus, posterior division L<br>1 voxels covering 0% of Cerebellum 6 R<br>42 voxels covering 0% of not-labeled
**6**	2	-20	-22	256	0.000002	<0.000001	149 voxels covering 4% of Brain-Stem<br>107 voxels covering 0% of not-labeled
**7**	34	-12	-36	168	0.000107	0.000002	60 voxels covering 8% of Temporal Fusiform Cortex, posterior division R<br>22 voxels covering 7% of Inferior Temporal Gyrus, anterior division R<br>20 voxels covering 3% of Parahippocampal Gyrus, anterior division R<br>10 voxels covering 3% of Temporal Fusiform Cortex, anterior division R<br>9 voxels covering 1% of Inferior Temporal Gyrus, posterior division R<br>47 voxels covering 0% of not-labeled
**8**	40	-62	-30	165	0.000125	0.000002	131 voxels covering 5% of Cerebellum Crus1 R<br>28 voxels covering 2% of Cerebellum 6 R<br>6 voxels covering 0% of Cerebellum Crus2 R
**9**	-32	-10	-42	141	0.000454	0.000008	72 voxels covering 8% of Temporal Fusiform Cortex, posterior division L<br>45 voxels covering 14% of Temporal Fusiform Cortex, anterior division L<br>6 voxels covering 1% of Inferior Temporal Gyrus, posterior division L<br>18 voxels covering 0% of not-labeled

**10**	18	20	4	133	0.00071	0.000013	116 voxels covering 22% of Caudate r<br>17 voxels covering 0% of not-labeled
**Frontoparietal 2**							
**1**	60	4	-28	600	<0.000001	<0.000001	246 voxels covering 18% of Middle Temporal Gyrus, posterior division R<br>88 voxels covering 21% of Superior Temporal Gyrus, posterior division R<br>83 voxels covering 20% of Middle Temporal Gyrus, anterior division R<br>25 voxels covering 9% of Superior Temporal Gyrus, anterior division R<br>21 voxels covering 1% of Temporal Pole R<br>137 voxels covering 0% of not-labeled
**2**	-44	22	-32	255	0.000001	<0.000001	193 voxels covering 8% of Temporal Pole L<br>35 voxels covering 8% of Middle Temporal Gyrus, anterior division L<br>9 voxels covering 3% of Superior Temporal Gyrus, anterior division L<br>18 voxels covering 0% of not-labeled
**3**	60	-22	44	131	0.000558	0.00001	108 voxels covering 13% of Supramarginal Gyrus, anterior division R<br>9 voxels covering 0% of Postcentral Gyrus R<br>14 voxels covering 0% of not-labeled
**4**	-54	-16	-12	98	0.004244	0.000074	41 voxels covering 3% of Middle Temporal Gyrus, posterior division L<br>6 voxels covering 2% of Superior Temporal Gyrus, anterior division L<br>3 voxels covering 1% of Superior Temporal Gyrus, posterior division L<br>2 voxels covering 0% of Middle Temporal Gyrus, anterior division L<br>46 voxels covering 0% of not-labeled
**Frontoparietal 1**							
**1**	12	-24	72	174	0.000043	0.000001	165 voxels covering 4% of Precentral Gyrus R<br>3 voxels covering 0% of Superior Frontal Gyrus R<br>6 voxels covering 0% of not-labeled
**2**	34	22	-6	164	0.000075	0.000001	88 voxels covering 6% of Frontal Orbital Cortex R<br>60 voxels covering 4% of Insular Cortex R<br>3 voxels covering 1% of Frontal Operculum Cortex R<br>13 voxels covering 0% of not-labeled
**3**	40	48	6	155	0.000123	0.000002	149 voxels covering 2% of Frontal Pole R<br>4 voxels covering 1% of Inferior Frontal Gyrus, pars triangularis R<br>1 voxels covering 0% of Middle Frontal Gyrus R<br>1 voxels covering 0% of not-labeled
**4**	38	-64	42	100	0.003379	0.000058	80 voxels covering 2% of Lateral Occipital Cortex, superior division R<br>8 voxels covering 1% of Angular Gyrus R<br>7 voxels covering 0% of Superior Parietal Lobule R<br>5 voxels covering 0% of not-labeled
**Executive Control**							
**1**	-6	-86	8	4712	<0.000001	<0.000001	482 voxels covering 18% of Occipital Pole L<br>461 voxels covering 9% of Lateral Occipital Cortex, superior division L<br>431 voxels covering 12% of Postcentral Gyrus L<br>372 voxels covering 58% of Cuneal Cortex R<br>331 voxels covering 52% of Intracalcarine Cortex L<br>308 voxels covering 6% of Lateral Occipital Cortex, superior division R<br>303 voxels covering 40% of Intracalcarine Cortex R<br>291 voxels covering 12% of Occipital Pole R<br>253 voxels covering 15% of Lingual Gyrus R<br>234 voxels covering 4% of Precuneous Cortex<br>225 voxels covering 15% of Lingual Gyrus L<br>213 voxels covering 41% of Cuneal Cortex L<br>115 voxels covering 8% of Superior Parietal Lobule L<br>95 voxels covering 66% of Supracalcarine Cortex R<br>47 voxels covering 2% of Cingulate Gyrus, posterior division<br>31 voxels covering 42% of Supracalcarine Cortex L<br>4 voxels covering 0% of Precentral Gyrus L<br>2 voxels covering 0% of Cerebellum 4 5 L<br>514 voxels covering 0% of not-labeled

**2**	36	-38	56	736	<0.000001	<0.000001	327 voxels covering 10% of Postcentral Gyrus R<br>243 voxels covering 16% of Superior Parietal Lobule R<br>127 voxels covering 3% of Precentral Gyrus R<br>39 voxels covering 0% of not-labeled
**3**	-8	-36	10	591	<0.000001	<0.000001	34 voxels covering 2% of Thalamus l<br>32 voxels covering 3% of Thalamus r<br>525 voxels covering 0% of not-labeled
**4**	12	-16	0	252	0.000002	<0.000001	236 voxels covering 19% of Thalamus r<br>16 voxels covering 0% of not-labeled
**5**	-10	-16	2	193	0.000035	0.000001	188 voxels covering 14% of Thalamus l<br>5 voxels covering 0% of not-labeled
**6**	22	38	-4	114	0.002352	0.000044	114 voxels covering 0% of not-labeled
**Visual 3**							
**1**	2	-100	10	251	0.000002	<0.000001	199 voxels covering 8% of Occipital Pole R<br>22 voxels covering 3% of Intracalcarine Cortex R<br>30 voxels covering 0% of not-labeled
**2**	38	-76	-30	201	0.000018	<0.000001	201 voxels covering 8% of Cerebellum Crus1 R
**3**	-10	-78	16	133	0.000646	0.000012	112 voxels covering 17% of Intracalcarine Cortex L<br>1 voxels covering 0% of Cuneal Cortex L<br>1 voxels covering 1% of Supracalcarine Cortex L<br>19 voxels covering 0% of not-labeled
**Auditory**							
**-**	-	-	-	-	-	-	-
**Sensorimotor**							
**1**	8	-10	48	9115	<0.000001	<0.000001	2158 voxels covering 50% of Precentral Gyrus R<br>1999 voxels covering 46% of Precentral Gyrus L<br>979 voxels covering 27% of Postcentral Gyrus L<br>831 voxels covering 26% of Postcentral Gyrus R<br>323 voxels covering 45% of Juxtapositional Lobule Cortex -formerly Supplementary Motor Cortex- R<br>213 voxels covering 33% of Juxtapositional Lobule Cortex -formerly Supplementary Motor Cortex- L<br>144 voxels covering 6% of Cingulate Gyrus, anterior division<br>96 voxels covering 3% of Superior Frontal Gyrus L<br>91 voxels covering 7% of Insular Cortex R<br>90 voxels covering 3% of Superior Frontal Gyrus R<br>80 voxels covering 9% of Central Opercular Cortex R<br>62 voxels covering 3% of Cingulate Gyrus, posterior division<br>30 voxels covering 6% of Parietal Operculum Cortex R<br>7 voxels covering 2% of Heschl's Gyrus R<br>1 voxels covering 0% of Precuneous Cortex<br>2011 voxels covering 1% of not-labeled
**2**	-36	-48	42	2173	<0.000001	<0.000001	587 voxels covering 62% of Supramarginal Gyrus, anterior division L<br>486 voxels covering 33% of Superior Parietal Lobule L<br>414 voxels covering 8% of Lateral Occipital Cortex, superior division L<br>253 voxels covering 7% of Postcentral Gyrus L<br>115 voxels covering 11% of Supramarginal Gyrus, posterior division L<br>81 voxels covering 1% of Precuneous Cortex<br>3 voxels covering 0% of Angular Gyrus L<br>234 voxels covering 0% of not-labeled
**3**	-20	-70	-22	820	<0.000001	<0.000001	423 voxels covering 33% of Cerebellum 6 L<br>336 voxels covering 15% of Cerebellum Crus1 L<br>27 voxels covering 4% of Temporal Occipital Fusiform Cortex L<br>4 voxels covering 0% of Occipital Fusiform Gyrus L<br>1 voxels covering 0% of Cerebellum Crus2 L<br>1 voxels covering 0% of Cerebellum 4 5 L<br>1 voxels covering 0% of Cerebellum 7b L<br>27 voxels covering 0% of not-labeled

**4**	26	-72	-22	810	<0.000001	<0.000001	485 voxels covering 31% of Cerebellum 6 R<br>278 voxels covering 11% of Cerebellum Crus1 R<br>16 voxels covering 1% of Cerebellum Crus2 R<br>14 voxels covering 7% of Vermis 7<br>9 voxels covering 3% of Vermis 6<br>3 voxels covering 0% of Temporal Occipital Fusiform Cortex R<br>2 voxels covering 0% of Occipital Fusiform Gyrus R<br>3 voxels covering 0% of not-labeled
**5**	38	-46	54	701	<0.000001	<0.000001	361 voxels covering 24% of Superior Parietal Lobule R<br>118 voxels covering 2% of Lateral Occipital Cortex, superior division R<br>78 voxels covering 6% of Supramarginal Gyrus, posterior division R<br>37 voxels covering 1% of Postcentral Gyrus R<br>8 voxels covering 1% of Angular Gyrus R<br>5 voxels covering 1% of Supramarginal Gyrus, anterior division R<br>94 voxels covering 0% of not-labeled
**6**	60	-18	22	419	<0.000001	<0.000001	198 voxels covering 25% of Supramarginal Gyrus, anterior division R<br>142 voxels covering 4% of Postcentral Gyrus R<br>6 voxels covering 0% of Supramarginal Gyrus, posterior division R<br>5 voxels covering 1% of Central Opercular Cortex R<br>68 voxels covering 0% of not-labeled
**7**	-38	32	12	392	<0.000001	<0.000001	154 voxels covering 2% of Frontal Pole L<br>96 voxels covering 3% of Middle Frontal Gyrus L<br>89 voxels covering 14% of Inferior Frontal Gyrus, pars triangularis L<br>53 voxels covering 0% of not-labeled
**8**	-44	8	40	360	<0.000001	<0.000001	253 voxels covering 9% of Middle Frontal Gyrus L<br>65 voxels covering 8% of Inferior Frontal Gyrus, pars opercularis L<br>24 voxels covering 1% of Precentral Gyrus L<br>18 voxels covering 0% of not-labeled
**9**	0	34	42	230	0.000008	<0.000001	61 voxels covering 2% of Superior Frontal Gyrus R<br>52 voxels covering 2% of Superior Frontal Gyrus L<br>40 voxels covering 3% of Paracingulate Gyrus L<br>12 voxels covering 1% of Paracingulate Gyrus R<br>65 voxels covering 0% of not-labeled
**10**	-32	-24	18	227	0.000009	<0.000001	72 voxels covering 5% of Insular Cortex L<br>65 voxels covering 7% of Central Opercular Cortex L<br>45 voxels covering 8% of Parietal Operculum Cortex L<br>6 voxels covering 2% of Heschl's Gyrus L<br>1 voxels covering 0% of Planum Temporale L<br>38 voxels covering 0% of not-labeled
**11**	46	40	12	211	0.000019	<0.000001	190 voxels covering 2% of Frontal Pole R<br>2 voxels covering 0% of Inferior Frontal Gyrus, pars triangularis R<br>1 voxels covering 0% of Middle Frontal Gyrus R<br>18 voxels covering 0% of not-labeled
**12**	44	8	36	197	0.000037	0.000001	118 voxels covering 4% of Middle Frontal Gyrus R<br>59 voxels covering 1% of Precentral Gyrus R<br>20 voxels covering 0% of not-labeled
**13**	-28	52	20	167	0.000162	0.000003	167 voxels covering 2% of Frontal Pole L
**14**	30	-2	-8	154	0.000316	0.000006	107 voxels covering 13% of Putamen r<br>6 voxels covering 2% of Amygdala r<br>41 voxels covering 0% of not-labeled
**15**	32	-74	-52	125	0.001504	0.000029	63 voxels covering 3% of Cerebellum 8 R<br>59 voxels covering 11% of Cerebellum 7b R<br>2 voxels covering 0% of Cerebellum Crus2 R<br>1 voxels covering 0% of not-labeled
**16**	-28	2	62	123	0.001682	0.000032	81 voxels covering 3% of Superior Frontal Gyrus L<br>31 voxels covering 1% of Middle Frontal Gyrus L<br>11 voxels covering 0% of not-labeled

**17**	-4	-48	-10	118	0.002229	0.000043	55 voxels covering 9% of Vermis 4 5<br>31 voxels covering 5% of Cerebellum 4 5 R<br>19 voxels covering 2% of Cerebellum 4 5 L<br>11 voxels covering 5% of Vermis 3<br>2 voxels covering 1% of Cerebellum 3 R
**18**	-28	-18	2	117	0.00236	0.000045	54 voxels covering 6% of Putamen l<br>4 voxels covering 1% of Amygdala l<br>3 voxels covering 1% of Pallidum l<br>56 voxels covering 0% of not-labeled
**19**	-12	4	12	114	0.002801	0.000054	60 voxels covering 11% of Caudate l<br>3 voxels covering 0% of Thalamus l<br>51 voxels covering 0% of not-labeled

Table 2

Cluster no.	MNI			Size (voxels)	p value FWE (cluster size)	p value uncorrected (cluster size)	Brain regions (percentage covered by cluster)
	x	y	z				
Increases in degree centrality (MDMA – placebo)							
1	-36<br>-44<br>-24	-40<br>-34<br>-42	68<br>58<br>72	230	<0.001	<0.001	Precentral Gyrus l (0.09%)<br>Postcentral Gyrus l (32.8%)<br>Superior Parietal Lobule l (24.4%)<br>Supramarginal Gyrus, anterior division l (1.8%)<br>Supramarginal Gyrus, posterior division l (0.6%)<br>Angular Gyrus l (<0.1%)
2	-6<br>8<br>-4	10<br>0<br>-4	6<br>12<br>14	163	<0.001	<0.001	Lateral Ventricle l (27.2%)<br>Thalamus l (6.1%)<br>Caudate l (24.1%)<br>Accumbens l (0.1%)<br>Cerebral White Matter r (6.5%)<br>Cerebral Cortex r (0.7%)<br>Lateral Ventricle r (12.2%)<br>Thalamus r (5.3%)<br>Caudate r (4.7%)<br>Accumbens r (0.2%)
3	46<br>34<br>36	12<br>14<br>6	-18<br>-28<br>-30	106	0.002	<0.001	Insular Cortex r (0.1%)<br>Temporal Pole r (55.2%)<br>Superior Temporal Gyrus, anterior division r (<0.1%)<br>Frontal Orbital Cortex r (0.7%)<br>Parahippocampal Gyrus, anterior division r (0.1%)<br>Temporal Fusiform Cortex, anterior division r (<0.1%)<br>Planum Polare r (1.9%)<br>Amygdala r (<0.1%)
4	-52<br>-52	-60<br>-64	2<br>-6	107	0.002	<0.001	Middle Temporal Gyrus, temporooccipital part l (25.1%)<br>Inferior Temporal Gyrus, temporooccipital part l (6.4%)<br>Supramarginal Gyrus, posterior division l (<0.1%)<br>Angular Gyrus l (0.1%)<br>Lateral Occipital Cortex, superior division l (<0.1%)<br>Lateral Occipital Cortex, inferior division l (30.7%)
5	40<br>36<br>40	-34<br>-42<br>-36	54<br>56<br>62	504	<0.001	<0.001	Precentral Gyrus r (0.1%)<br>Postcentral Gyrus r (31.4%)<br>Superior Parietal Lobule r (18.5%)<br>Supramarginal Gyrus, anterior division r (9.4%)<br>Supramarginal Gyrus, posterior division r (3.1%)<br>Angular Gyrus r (0.1%)<br>Central Opercular Cortex r (<0.1%)<br>Parietal Operculum Cortex r (0.2%)<br>Planum Temporale r (<0.1%)
6	2<br>-4<br>2	-38<br>-26<br>-26	-38<br>-30<br>-42	235	<0.001	<0.001	Brain-Stem:92.3490<br>Cerebellum (L IX: 1.5%, Vermis IX: 0.1%, R IX: 1.7%, L X: 0.1%, Vermis X: 0.9%, R X: 0.1%)
7	-48<br>-50	-26<br>-28	36<br>50	115	0.001	<0.001	Postcentral Gyrus l (44.0%)<br>Superior Parietal Lobule l (1.9%)<br>Supramarginal Gyrus, anterior divisionl (19.5%)

**8**	-28<br>-36<br>-38	-4<br>-2<br>2	64<br>60<br>50	132	<0.001	<0.001	Superior Frontal Gyrus l (1<0.1%)<br>Middle Frontal Gyrus l (21.1%)<br>Precentral Gyrus l (25.6%
**9**	50<br>50<br>54	-48<br>-58<br>-40	-20<br>-18<br>-22	78	0.014	<0.001	Middle Temporal Gyrus, temporooccipital part r (<0.1%)<br>Inferior Temporal Gyrus, posterior division r (2.1%)<br>Inferior Temporal Gyrus, temporooccipital part r (49.4%)<br>Lateral Occipital Cortex, inferior division r (9.0%)<br>Temporal Fusiform Cortex, posterior division r (0.5%)<br>Temporal Occipital Fusiform Cortex r (15.6%)<br>Occipital Fusiform Gyrus r (1.5%)<br>Cerebellum (R Crus I: 0.2%)
**10**	38<br>48<br>54	6<br>10<br>14	24<br>28<br>20	83	0.01	<0.001	Middle Frontal Gyrus r (6.6%)<br>Inferior Frontal Gyrus, pars opercularis r (21.4%)<br>Precentral Gyrus r (28.0%)
**11**	28<br>22	0<br>-6	62<br>52	78	0.014	<0.001	Superior Frontal Gyrus r (25.7%)<br>Middle Frontal Gyrus r (13.7%)<br>Precentral Gyrus (12.1%)
Decreases in degree centrality (MDMA – placebo)							
**1**	-20<br>-14<br>-10	-96<br>-86<br>-94	24<br>22<br>26	621	<0.001	<0.001	Lateral Occipital Cortex, superior division l (6.0%)<br>Lateral Occipital Cortex, inferior division l (1.0%)<br>Intracalcarine Cortex l (0.4%)<br>Precuneous Cortex l (0.4%)<br>Cuneal Cortex l (6.2%)<br>Supracalcarine Cortex l (0.6%)<br>Occipital Pole l (41.2%)
**2**	-4<br>-12<br>-14	-64<br>-62<br>-54	-4<br>-14<br>-16	99	0.003	<0.001	Inferior Temporal Gyrus, temporooccipital part l (<0.1%)<br>Intracalcarine Cortex l (<0.1%)<br>Lingual Gyrus l (27.9%)<br>Temporal Occipital Fusiform Cortex l (1.2%)<br>Occipital Fusiform Gyrus l (0.6%)<br>Cerebellum (L I-IV: 2.4%, L V: 29.9%, L VI: 19.0%, Vermis VI: 0.3%)
**3**	14<br>28<br>18	-82<br>-58<br>-92	28<br>12<br>34	566	<0.001	<0.001	Lateral Occipital Cortex, superior division r (7.5%)<br>Lateral Ventricle r (1.2%)<br>Lateral Occipital Cortex, inferior division r (0.1%)<br>Intracalcarine Cortex r (4.5%)<br>Cingulate Gyrus, posterior division r (<0.1%)<br>Precuneous Cortex r (3.6%)<br>Cuneal Cortex r (9.5%)<br>Lingual Gyrus r (0.7%)<br>Occipital Fusiform Gyrus r (<0.1%)<br>Supracalcarine Cortex r (1.5%)<br>Occipital Pole r (24.1%)
**4**	40<br>8<br>22	-70<br>-60<br>-66	-30<br>-8<br>-18	818	<0.001	<0.001	Lateral Occipital Cortex, inferior division r (0.3%)<br>Intracalcarine Cortex R (<0.1%)<br>Cingulate Gyrus, posterior division r (<0.1%)<br>Precuneous Cortex r (0.01%)<br>Lingual Gyrus r (8.5%)<br>Temporal Occipital Fusiform Cortex r (0.5%)<br>Occipital Fusiform Gyrus r (1.7%)<br>Occipital Pole r (<0.1%)<br>Cerebellum (R I-IV: 0.5%, R V:4.2%, L VI: 0.2%, Vermis VI: 3.5%, R VI: 17.8%, L Crus I: 0.3%, Vermis Crus I: 0.2%, R Crus I: 39.7%, L Crus II: 0.9%, Vermis Crus II: 0.1%, R Crus II: 9.2%, R VIIb: 0.2%)
**5**	-34<br>-34<br>-34	-78<br>-72<br>-68	-26<br>-32<br>-24	349	<0.001	<0.001	Inferior Temporal Gyrus, temporooccipital part l (<0.1%)<br>Lateral Occipital Cortex, inferior division l (0.5%)<br>Lingual Gyrus l (0.2%)<br>Temporal Occipital Fusiform Cortex l (0.1%)<br>Occipital Fusiform Gyrus l (1.9%)<br>Occipital Pole l (<0.1%)<br>Cerebellum (L VI: 8.1%, L Crus I: 79.4%, Vermis Crus I: <0.1%, L Crus II: 3.8%)

References

1. Smith SM, Fox PT, Miller KL, Glahn DC, Fox PM, Mackay CE, et al. Correspondence of the brain’s functional architecture during activation and rest. Proc Natl Acad Sci U S A. 2009;106:13040–13045.
2. Carhart-Harris RL, Muthukumaraswamy S, Roseman L, Kaelen M, Droog W, Murphy K, et al. Neural correlates of the LSD experience revealed by multimodal neuroimaging. Proc Natl Acad Sci U S A. 2016;113:4853–4858.
3. Müller F, Dolder PCPC, Schmidt A, Liechti MEME, Borgwardt S. Altered network hub connectivity after acute LSD administration. NeuroImage Clin. 2018;18:694–701.
4. Desikan RS, Ségonne F, Fischl B, Quinn BT, Dickerson BC, Blacker D, et al. An automated labeling system for subdividing the human cerebral cortex on MRI scans into gyral based regions of interest. Neuroimage. 2006. 2006. https://doi.org/10.1016/j.neuroimage.2006.01.021.
5. Tzourio-Mazoyer N, Landeau B, Papathanassiou D, Crivello F, Etard O, Delcroix N, et al. Automated anatomical labeling of activations in SPM using a macroscopic anatomical parcellation of the MNI MRI single-subject brain. Neuroimage. 2002. 2002. https://doi.org/10.1006/nimg.2001.0978.
