# Supplementary material for: MDMA-induced changes in within-network connectivity contradict the specificity of these alterations for the effects of serotonergic hallucinogens
Source: Neuropsychopharmacology. 2020 Nov 20;46(3):545–53. doi: 10.1038/s41386-020-00906-2 (PMC8027447; doi:10.1038/s41386-020-00906-2)
Supplement: Supplementary file 3 — CONSORT flowchart [file 41386_2020_906_MOESM3_ESM.pptx]

## Slide 1
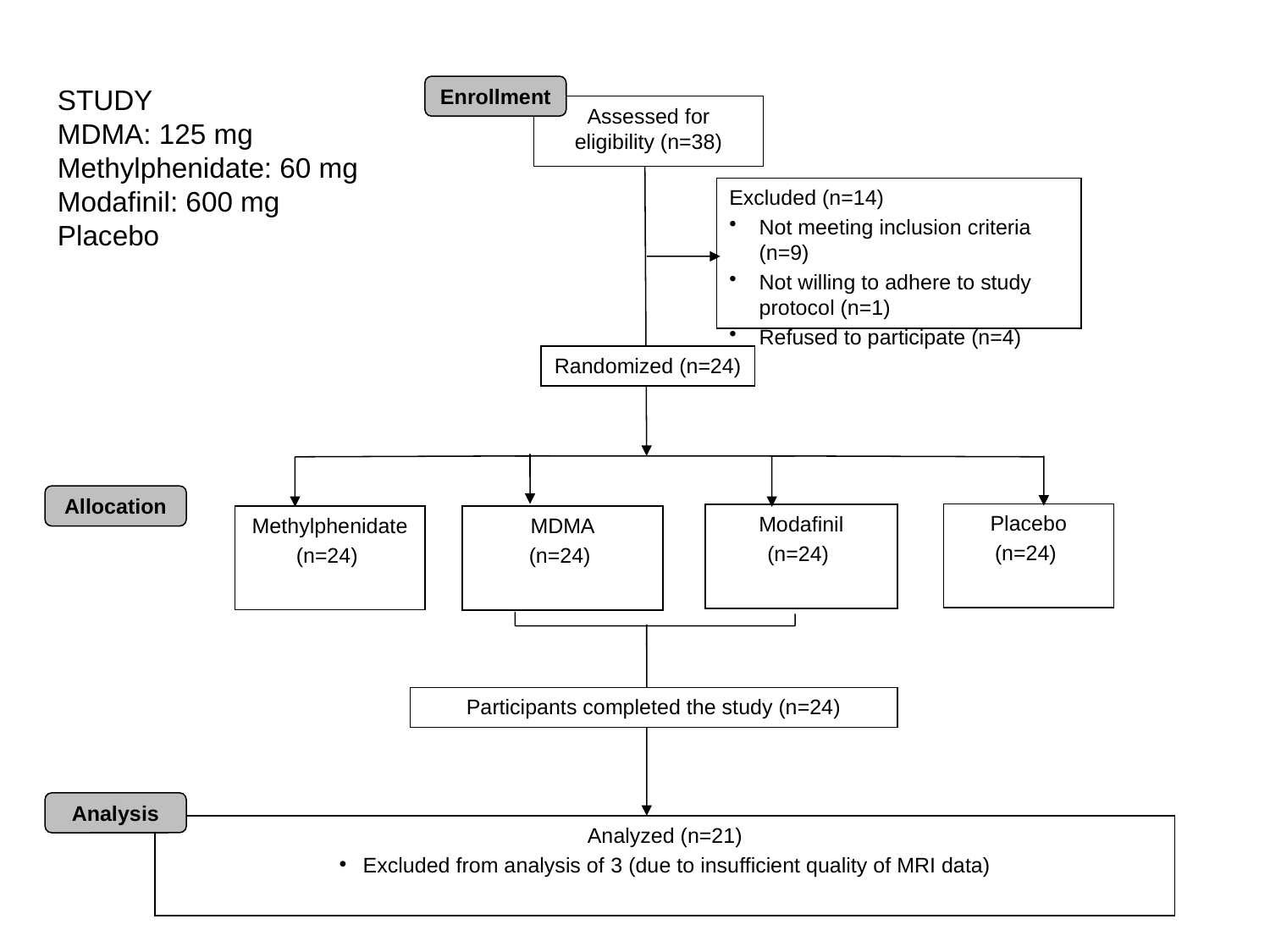

STUDY
MDMA: 125 mg
Methylphenidate: 60 mg
Modafinil: 600 mg
Placebo
Enrollment
Assessed for eligibility (n=38)
Excluded (n=14)
Not meeting inclusion criteria (n=9)
Not willing to adhere to study protocol (n=1)
Refused to participate (n=4)
Randomized (n=24)
Allocation
Placebo
(n=24)
Modafinil
(n=24)
Methylphenidate
(n=24)
MDMA
(n=24)
Participants completed the study (n=24)
Analysis
Analyzed (n=21)
Excluded from analysis of 3 (due to insufficient quality of MRI data)
